# ERK phosphorylation functions in invadopodia formation in tongue cancer cells in a novel silicate fibre-based 3D cell culture system

**DOI:** 10.1038/s41368-018-0033-y

**Published:** 2018-10-22

**Authors:** Masaharu Noi, Ken-Ichi Mukaisho, Saori Yoshida, Shoko Murakami, Shinya Koshinuma, Takeshi Adachi, Yoshisato Machida, Masashi Yamori, Takahisa Nakayama, Gaku Yamamoto, Hiroyuki Sugihara

**Affiliations:** 1 Department of Oral and Maxillofacial Surgery, Shiga University of Medical Science, Ōtsu, Shiga Japan; 2 Department of Pathology, Division of Molecular Diagnostic Pathology, Shiga University of Medical Science, Ōtsu, Shiga Japan; 3 Dental Oral Surgery, Nagahama Red Cross Hospital, Nagahama, Shiga Japan

## Abstract

To screen for additional treatment targets against tongue cancer, we evaluated the contributions of extracellular signal-related kinase (ERK), AKT and ezrin in cancer development. Immunohistochemical staining showed that ERK and ezrin expressions were significantly higher in invasive squamous cell carcinoma than in carcinoma in situ. To investigate the roles of ERK and ezrin in cancer development, we used the non-woven silica fibre sheet Cellbed^TM^ with a structure resembling the loose connective tissue morphology in a novel 3D culture system. We confirmed that the 3D system using Cellbed^TM^ accurately mimicked cancer cell morphology in vivo. Furthermore, cell projections were much more apparent in 3D-cultured tongue cancer cell lines than in 2D cultures. Typically, under conventional 2D culture conditions, F-actin and cortactin are colocalized in the form of puncta within cells. However, in the 3D-cultured cells, colocalization was mainly observed at the cell margins, including the projections. Projections containing F-actin and cortactin colocalization were predicted to be invadopodia. Although suppressing ezrin expression with small interfering RNA transfection caused no marked changes in morphology, cell projection formation was decreased, and the tumour thickness in vertical sections after 3D culture was markedly decreased after suppressing ERK activity because both the invasion ability and proliferation were inhibited. An association between cortactin activation as well as ERK activity and invadopodia formation was detected. Our novel 3D culture systems using Cellbed™ are simple and useful for in vitro studies before conducting animal experiments. ERK contributes to tongue cancer development by increasing both cancer cell proliferation and migration via cortactin activation.

## Oncology: ERK contribution to tongue cancer development

Extracellular signal-regulated kinases (ERKs) are enzymes that are involved in a variety of cell functions, and one in vitro study suggests that ERKs play a role in tongue cancer development by increasing cancer cell proliferation and migration. Using a novel 3-D cell culture system called Cellbed to mimic cancer cell morphology, a team headed by Ken-ichi Mukaisho at Shiga University of Medical Science, Japan found that ERKs activate cortactin (a protein located in the cell cytoplasm) and contribute to the formation of invadopodia (invasive cell protrusions associated with cancer cells) in tongue cancer cells and tumor development. The authors conclude that experimental 3-D cell culture systems employing Cellbed are easily implemented and useful for in vitro studies before conducting animal experiments and that they can be widely applied in cancer research.

## Introduction

Oral cancer ranks 15th worldwide in both morbidity and mortality.^1,2^ In Japan, the number of patients with oral cancer has been increasing each year; oral cancer develops most frequently in the tongue.^3^ To improve the prognosis of advanced tongue cancer, it is necessary to determine the molecular mechanisms associated with its development and develop new targeted treatments. We previously reported that ezrin contributes to the development of tongue cancer, suggesting its usefulness as a novel therapeutic target.^4^ To screen for additional treatment targets, we first evaluated the possible contributions of extracellular signal-related kinase (ERK) and AKT to the development of tongue cancer by immunohistochemical analyses. We found that ERK and ezrin were significantly overexpressed in invasive squamous cell carcinoma (SCC) compared to carcinoma in situ (CIS). Although it has been reported that AKT is associated with the progression of tongue cancer, AKT staining showed no significant difference in the degree of protein expression between CIS and SCC samples in our study. These results suggest that both ERK and ezrin contribute to the development of tongue cancer.

Most studies in the field of cancer research have been carried out with two-dimensional (2D) cultures in in vitro experimental systems using cancer cell lines; however, the 2D culture environment on the surface of hard tissue culture plates composed of polystyrene or glass considerably differs from the microenvironment within the body for basic activities.^5–8^ Therefore, experimental systems using 2D culture may not accurately reproduce the physiological effects of cancer cells in vivo.^9^ When cells isolated from tissues are subjected to 2D culture on a planar culture support, many cells become progressively flatter, divide abnormally, and lose their differentiated phenotype.^10,11^ Recently, increased attention has been given to mimicking the environment surrounding tumour cells in vivo, which is characterized by the abnormal accumulation of extracellular matrix components or key enzymes, the development of abnormal angiogenesis, and the incorporation of heterogeneous cell populations to investigate the physiological actions of tumour cells. In the current study, a novel 3D culture support composed of a fine non-woven silica fibre sheet was used as a scaffold. Cells cultured in this system with the silica fibre scaffold developed a 3D configuration more closely resembling cells, and thus accurately mimicking the morphology of tumour cells in vivo and promoting cell growth.^12^ We recently found that the shape of a Cellbed^TM^ resembles loose connective tissues in a living body.^13^ Moreover, podia formed more easily in this 3D system than in a 2D system.^13^

Invadopodia are actin-based membrane projections that cause the localized degradation of the extracellular matrix through the action of proteolytic enzymes; they are 0.1 μm–0.8 μm in diameter with a length of nearly 2 μm and play an important role in the invasion of surrounding tissue.^14–16^ Epithelial growth factor and ERK have been reported to contribute to invadopodia formation.^17^ Cortactin is a marker of invadopodia, and the colocalization of cortactin with F-actin indicates invadopodia formation.^18,19^

In this study, we investigated the role of ezrin and ERK in cancer development and determined whether these markers can be used as molecular targets. We suppressed the expression of ezrin and ERK and evaluated the changes in cancer cell behaviour and morphology, particularly invadopodia formation, by culturing tongue cancer cell lines in a novel 3D system using Cellbed^TM^.

## Results

### Screening of target proteins

#### Protein expression in human tongue tumour tissue

**Fig. 1:**
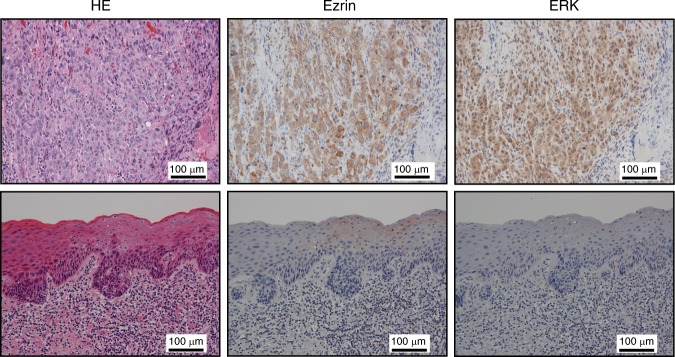
Representative images of ezrin and ERK staining of human tongue tumour tissue (scale bar: 100 μm). Upper: SCC (T1, T2) cases, lower: CIS cases. The brown colour represents cells positive for the proteins as visualized by DAB staining. The invasive part of tongue cancer cells was strongly positive for both proteins in SCC samples compared to CIS samples

The results of staining with each antibody are shown in Table 1. Staining for ezrin protein showed a high expression level in two cases and a low level in ten cases for CIS samples, as well as a high level in 12 cases and low level in one case for SCC samples (*P* = 0.0002). Staining for the ERK protein showed a high expression level in one case and a low level in 11 cases for CIS samples, but a high level in 11 cases and low level in two cases for SCC samples (*P* = 0.0002). Staining for the AKT protein showed a high expression level in one case and a low level in 11 cases for CIS samples, as well as a high level in six cases and a low level in seven cases for SCC samples. Thus, AKT staining showed no significant difference in the degree of protein expression between CIS and SCC samples (*P* = 0.073). The number of samples showing high expression levels of ezrin and ERK was significantly greater for SCC than for CIS. Fig. 1 shows the representative images of ezrin and ERK staining for CIS and SCC samples. The intensity of both the proteins of ezrin and ERK was significantly higher in SCC samples than in CIS samples.

**Table 1:** The degree of expression of different proteins in human tongue tumour tissue

	Erzin		ERK		AKT	
CIS *n* = 12	High	Low	High	Low	High	Low
	2	10	1	11	1	11
SSC *n* = 13	High	Low	High	Low	High	Low
	12	1	11	2	6	7
	*P* = 0.0002		*P* = 0.0002		*P* = 0.073	

There was no significant difference in AKT protein expression between CIS and SCC samples; a significant difference was observed for ezrin and ERK expression. The level of significance was *P* < 0.05

### Verification of a novel 3D culture system using Cellbed

#### 3D culture using a non-woven silica fibre sheet (Cellbed)

**Fig. 2:**
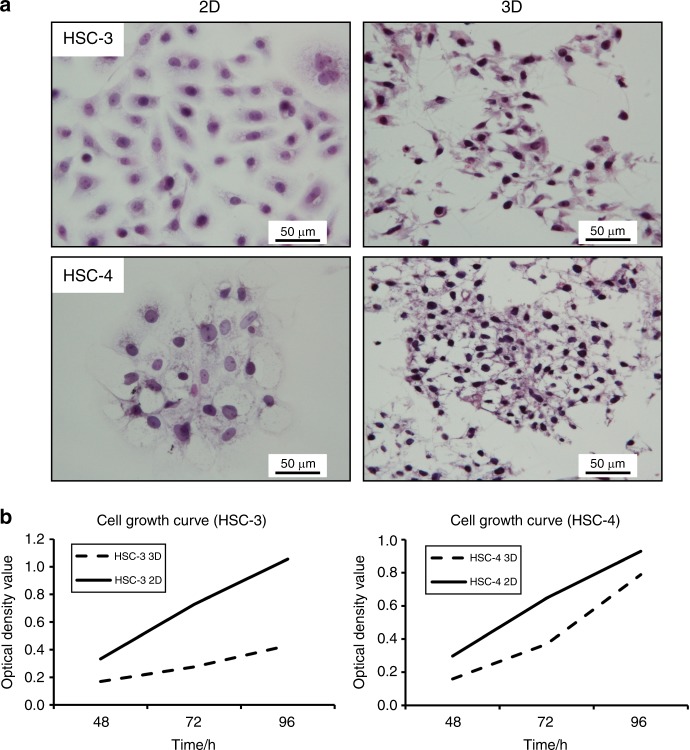
Differences in the morphology of 2D- and 3D-cultured HSC-3 and HSC-4 cells. **a** Cells cultured for 3 days on glass coverslips were mostly rounded. In contrast, protrusions were noted in the margin of cells 3D-cultured on Cellbed. HE staining (×400; scale bar: 50 μm). **b** MTT solution was added to the medium after incubation for 48, 72 or 96 h, and absorption was measured at each time point. We confirmed cell survival by MTT assay in both 2D and 3D culture conditions

**Fig. 3:**
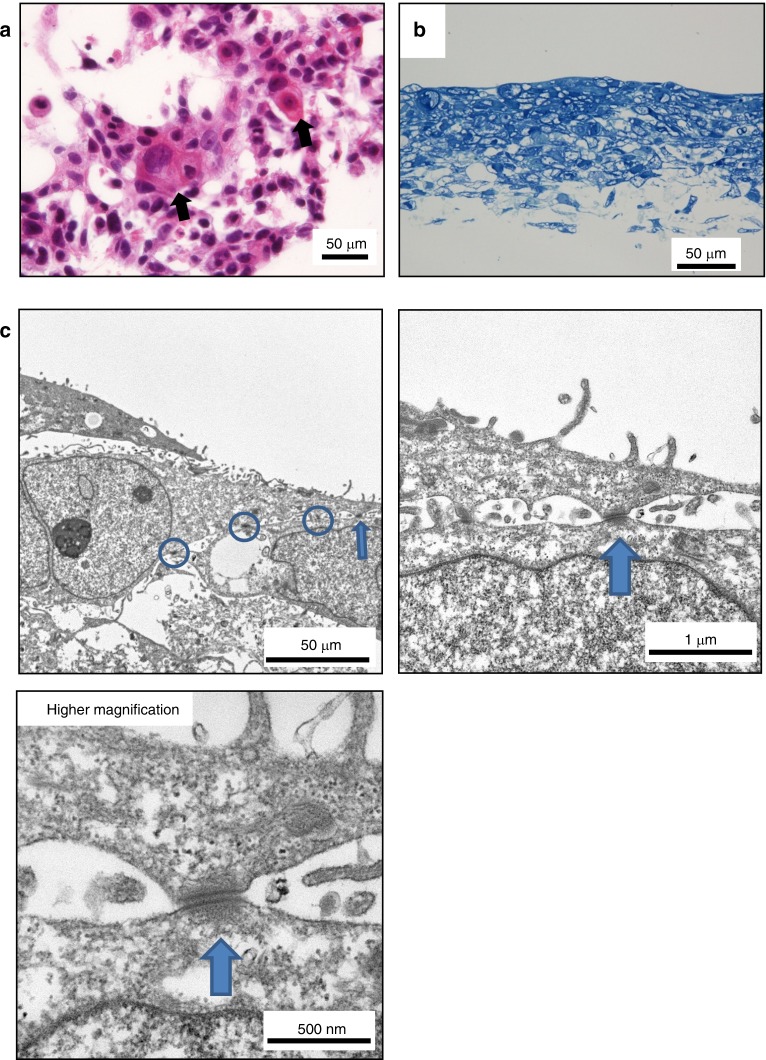
Cell morphology following long-term 3D culture. **a** Horizontal sections of Cellbed cultured with HSC-4 cells. Abnormal keratinization of SCC was observed (black arrow) (scale bar: 50 μm). **b** Vertical sections of long-term 3D-cultured HSC-4 cells. Toluidine blue-stained vertical section of HSC-4 cells 3D-cultured for approximately 4 weeks (scale bar: 50 μm). **c** Electron micrograph obtained after 3D culture for approximately 4 weeks. Desmosomes were detected in the vertical section of long-term 3D-cultured HSC-4 cells (arrows)

We compared the morphology of 3D- and 2D-cultured cells for HSC-3 and HSC-4 cell lines by haematoxylin and eosin (HE) staining. The 2D-cultured HSC-3 and HSC-4 cells appeared rounded. However, we easily detected cell projections in 3D-cultured HSC-3 and HSC-4 cells (Fig. 2a). We confirmed cell growth by MTT assay under both 2D and 3D culture conditions (Fig. 2b). In HSC-4 cells that had been 3D-cultured for a long period of approximately 4 weeks, abnormal keratinization of SCC was observed in the horizontal sectional images of specimens prepared parallel to the Cellbed (Fig. 3a). A layered structure of squamous epithelial cells was also observed in the vertical cross-sectional images of toluidine blue staining (Fig. 3b). Electron microscopy images of the verification of HSC-4 cells 3D-cultured for approximately 4 weeks also revealed structures that were found scattered over the lateral surface of epithelial cells. The structures have the characteristics of desmosomal attachments between cells (Fig. 3c). These findings demonstrated that the morphology of cells cultured using Cellbed was similar to their in vivo 3D structure.

### The roles of ERK and ezrin in cancer development

#### Evaluation of invasive capacity in 3D culture: a study of vertical sections

**Fig. 4:**
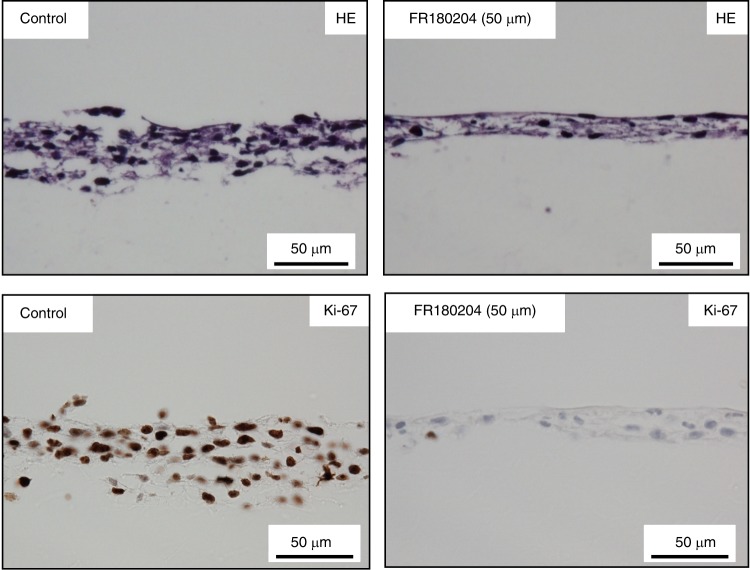
Forty-eight hours after HSC-4 cells were seeded into a 12-well plate, the medium was replaced with medium containing FR180204 (50 μmol•L^−1^). After 48 h, the medium was changed again to a medium containing FR180204 and incubated for another 48 h. Changes in the structure of squamous epithelial cancer cell layers following ERK inhibitor treatment. Suppression of the invasive capacity of HSC-4 cells was observed, with thinning of the layered structure of cancer cells compared to control cells, following the administration of the ERK inhibitor FR180204 (scale bar: 50 μm)

HE specimens were prepared from 3D-cultured HSC-4 cells given ERK inhibitor (FR180204) in the sections vertical to the Cellbed and were observed. The results showed that both untreated control cells (HSC-4C) and cells administered FR180204 (HSC-4FR) formed a layered structure based on squamous epithelial cells. We measured the width of infiltration of cancer cells in the vertical section as an index of invasion ability. We found that the layer thickness was thinner for HSC-4FR cells than for HSC-4C cells (Fig. 4). Additionally, we performed immunohistochemical staining of Ki67 and compared the Ki67-labelling index between FR and control cells. The Ki-67 index was much higher in control cells than in the FR cells (Fig. 4). These findings indicated that not only the invasive but also the proliferative capacity of HSC-4 cells was suppressed by FR180204.

#### Morphological changes in cancer cells following ezrin and ERK inhibition

**Fig. 5:**
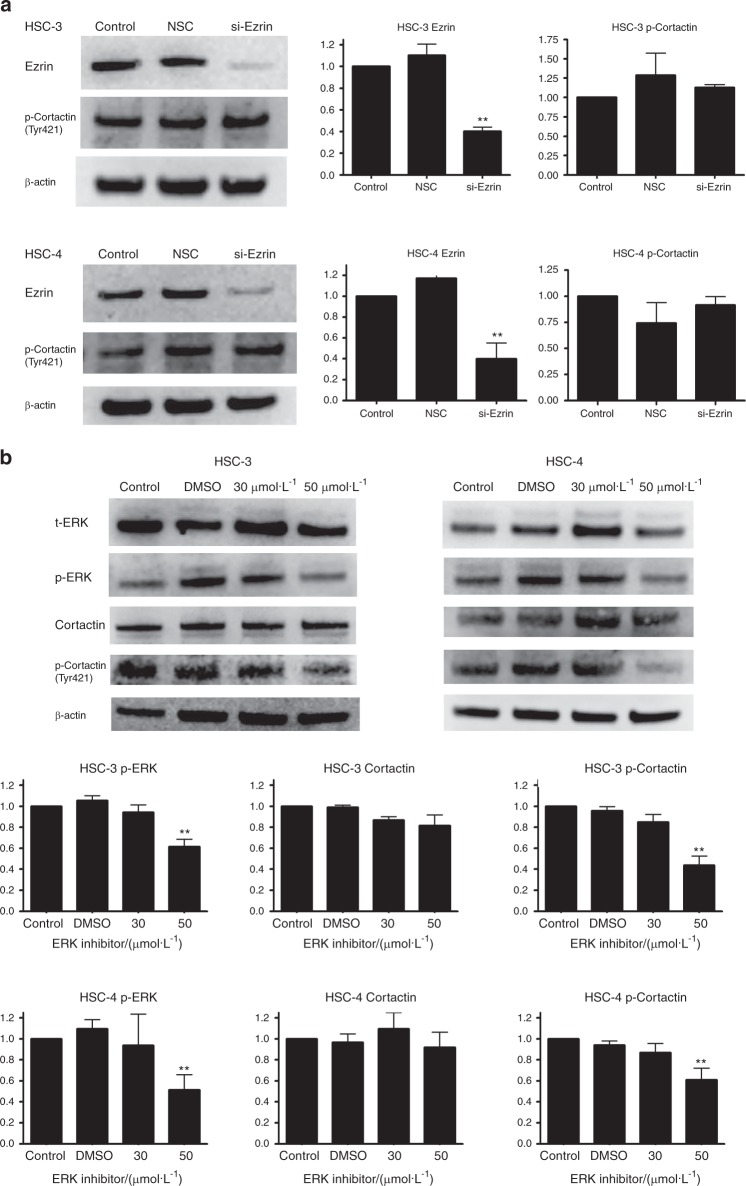
The effect of ezrin small interfering RNA (siRNA) and administration of ERK inhibitor (FR180204) on 3D-cultured HSC-3 and HSC-4 cells. **a** Expression of ezrin and p-cortactin (Tyr421) after siRNA treatment. Suppression of ezrin expression was observed in siEzrin cells in 3D-cultured HSC-3 and HSC-4 cell lines, but no difference was observed in the expression of p-cortactin (Tyr421) (*n* = 3). **b** Expression of p-ERK and cortactin and p-cortactin (Tyr421) following treatment with ERK inhibitor. In both 3D-cultured HSC-3 and HSC-4 cell lines, p-ERK expression was suppressed in FR cells, but no difference was observed in the expression of t-ERK. In addition, in FR cells, although no difference was observed in the expression of cortactin, p-cortactin (Tyr421) expression was significantly suppressed (*n* = 3)

The effect of ezrin small interfering RNA (siRNA) and administration of ERK inhibitor (FR180204) on 3D-cultured HSC-3 and HSC-4 cells was evaluated by western blotting by detecting the ezrin and phosphorylation-ERK (p-ERK) protein levels (Fig. 5).

**Fig. 6:**
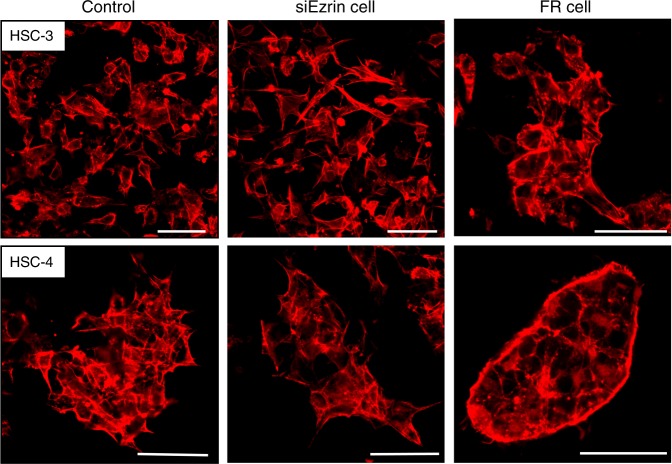
Changes in morphology following siRNA treatment (immunofluorescence staining). In both the tongue cancer cell lines (3D-cultured HSC-3 and HSC-4), cell projections formed in the control and siEzrin cells, while in FR cells, the formation of cell projections was inhibited (scale bar: 50 μm)

In both 3D-cultured HSC-3 and HSC-4 cells, the cytoskeleton was visualized by the immunofluorescent staining of F-actin after transfection with ezrin siRNA and treatment with ERK inhibitor. The results showed that the formation of cell projections was inhibited in FR cells compared to control cells, with a change in morphology to a rounded cobblestone shape, but no marked differences were observed in siEzrin cells (Fig. 6).

#### Relationship between ERK and cortactin in the regulation of cell morphology

We suspected that an association between ERK and the actin control protein, cortactin, was a causal factor in the changes in cell morphology due to the administration of ERK inhibitor (FR180204). To investigate the association between ERK and cortactin, changes in the expression levels of cortactin and p-cortactin (Tyr421) after the administration of FR180204 were investigated by western blotting. The results showed that in both 3D-cultured HSC-3 and HSC-4 cells, there were no changes in cortactin and p-cortactin (Tyr421) levels in siEzrin cells compared to non-silencing control cells (Fig. 5a), but the expression of p-cortactin was suppressed in 3D-cultured FR cells compared to control cells (Fig. 5b).

#### Evaluation of cancer cell invadopodia in the 3D culture environment

**Fig. 7:**
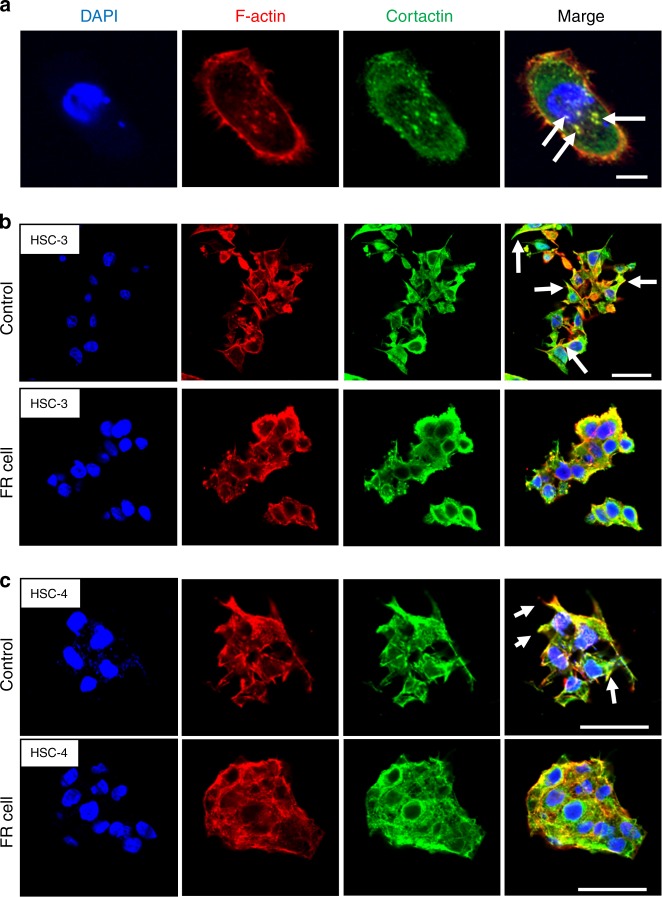
Immunofluorescent staining of 2D and 3D-cultured tongue cell lines. **a** Immunofluorescent staining was carried out on HSC-3 cells after 2D culture for 2 days on a glass coverslip. The colocalization of F-actin and cortactin, known as invadopodia, was observed in the form of punctae in the cell cytoplasm (scale bar: 10 μm). **b**, **c** Immunofluorescent staining of 3D-cultured tongue cell lines. Cell projections formed in the control cells in both HSC-3 (**b**) and HSC-4 cell lines (**c**); F-actin and cortactin colocalized in the cell margin. The formation of cell projections was markedly decreased in FR cells (scale bar: 50 μm)

Morphological changes in 3D-cultured tongue cancer cells (HSC-3 and HSC-4) following the administration of ERK inhibitor (FR180204) were evaluated by immunofluorescence staining, particularly to determine the sites of F-actin and cortactin colocalization. Ordinarily, under conventional 2D culture conditions, F-actin and cortactin are colocalized in the form of puncta within the cells (Fig. 7a). However, in 3D-cultured control cells, colocalization was mainly observed at the cell margins and was particularly noticeable (Fig. 7b, c). Moreover, cell projection formation was decreased by the administration of ERK inhibitor (FR180204), and colocalization was noted in a circle around the cytoskeleton. Based on these results, the projections in HSC-3 and HSC-4 cells observed in the 3D culture environment were predicted to be invadopodia.

## Discussion

A new experimental system involving 3D culture using the Cellbed, which accurately mimics the morphology of tumour cells in vivo, suggested that p-ERK activates cortactin and contributes to the formation of invadopodia in tongue cancer cells and tumour development.

Silver impregnation staining of human tongue tissue in this study revealed that the morphology of Cellbed resembles that of loose connective tissue with irregularly arranged collagen and reticular fibres. The mean flow pore diameter of Cellbed measured by the bubble point method on the horizontal surface of a single sheet was 7 μm–8 μm, but the pore diameter may be slightly wider in the actual 3D configuration. The normal size of a cell nucleus is approximately 3 μm–10 μm, while in cancer cells, the nucleus may be larger than 10 μm. In connective tissue in vivo, cancer cells are thought to proliferate and invade using collagen fibres as a scaffold or skeleton.^20^ In this study, vertical cross-sectional images of long-term 3D-cultured HSC-4 cells revealed that the squamous epithelium had a layered structure and differentiation towards the surface layer was confirmed. Abnormal keratinization of squamous epithelial carcinoma was also observed. Moreover, vertically oriented desmosomes were detected by electron microscopy. Based on these findings, the 3D culture system using Cellbed accurately mimicked the morphology of cancer cells in vivo. Thus, cancer cells 3D-cultured in Cellbed showed a layered structure and exhibited differentiation potential, possibly because Cellbed resembles the basic structure of loose connective tissue in vivo.

The Cellbed 3D culture system shows advantages in cancer research compared to other 3D culture systems. Various 3D culture systems have been developed, including standard 3D Matrigel culture, scaffolds using collagen or hyaluronic acid,^21,22^ and synthetic poly(lactide-co-glycolide), as well as methods employing cells-in-gels-in-paper^23,24^ or a microfluidic-based 3D culture device.^17^ Although currently widely used, Matrigel is derived from animals or cultured cells, and the quantity of the soluble structural components changes during experiments, decreasing the experimental reproducibility and reliability.^25^ The application of other systems is limited because of difficulties in reproducing 3D culture and controlling the 3D structure;^26^ the culture method is complex and the long-term culture or extraction of constituents such as proteins is difficult. In contrast, the Cellbed culture method is simple; it is conducted in a conventional culture plate and long-term culture for approximately 4 weeks and protein extraction is possible. Moreover, Cellbed can be cut after cell culture and used in other experiments. Thus, Cellbed is useful as a 3D culture support because it can be used in experiments that are difficult to perform using other 3D culture systems.

ERK is a downstream component of the signalling module activated by Raf serine/threonine kinase. In cancer, it causes the abnormal activation of ERK/mitogen-activated protein kinase (MAPK) signalling following upstream activation, particularly by epidermal growth factor receptor and Ras GTPases (small guanosine triphosphatases), and promotes the proliferation and survival of cancer cells and metastasis.^27^ Although Theocaris et al. reported that the detection of total-ERK (t-ERK) and p-ERK1/2 expression in infiltrating lymphoid cells of mobile tongue cancer is much more informative than measuring their expression in cancer cells,^28^ Degen et al. found that ERK plays an important role in the oral epithelium prior to tumour invasion;^29^ our results are consistent with these findings. To examine the roles of ERK in invadopodia formation associated with cancer development, we administered the ERK inhibitor FR180204 to HSC-3 and HSC-4 cells in both the 2D and 3D culture systems. Under 2D culture conditions, the colocalization of cortactin and F-actin was previously reported to be observed in the form of puncta within cells. However, in the present study using 3D culture, colocalization was strong at the margins of cell projections. Furthermore, the ERK inhibitor decreased cell projection formation and altered the cell morphology. Although we did not examine whether the projections can degrade the extracellular matrix, the present results suggest that in vivo invadopodia are present within the cell projections, which contributes to cancer invasion, rather than in the form of puncta in the cytoplasm, as observed under 2D culture conditions.

Cortactin is an F-actin-binding protein localized in the cell margin where actin reconstruction occurs, which is necessary for cell migration in the basal state. It is thought to act as a link between the cytoplasm and nucleus.^30^ ERK is known to be associated with cortactin activation^31,32^ and has been reported to be associated with the phosphorylation of S405 and S418 in cortactin.^33,34^ In the present study, analysis of cortactin phosphorylation at Tyr421 revealed the greater suppression of p-cortactin (Tyr421) in FR cells than in control cells. No previous studies have reported the relationship between ERK and Tyr421 and the details remain unclear; however, based on the results of the present study, p-cortactin (Tyr421) is associated with the formation of cell projections as well as ERK and cortactin signalling, which activates Tyr421.

In summary, our results suggest that cortactin is activated by p-ERK and contributes to invadopodia formation and tongue cancer development based on experiments performed in a novel 3D culture system using Cellbed. Thus, experimental 3D culture systems using Cellbed are simple and useful for in vitro studies before conducting animal experiments and can be widely applied in cancer research.

## Materials and methods

### Screening of target proteins

#### Human tongue cancer tissue

Formalin-fixed paraffin embedded block specimens of CIS (12 cases) and invasive SCC of the tongue (T1: ten cases, T2: three cases), surgically removed at the Shiga University of Medical Science hospital, were examined in this study. This study was approved by the Ethics Committee of the hospital (Approval No. 27-78).

#### Immunohistochemical staining of human tongue cancer tissue

To screen for the target proteins, we first evaluated the possible contributions of ezrin, ERK and AKT by the immunohistological staining of human tongue cancer tissues. Three-micrometre-thick sequential sections were prepared from the paraffin blocks of the maximum cut surface specimens for each case. The intensity of staining was classified on a four-point scale: 0, 1+, 2+, 3+, with 0 and 1+, indicating low levels and 2+ and 3+, indicating high levels. The degree of staining was compared between CIS and SCC samples. The antibodies used were as follows: anti-ezrin rabbit antibody (1:100, #3145; Cell Signalling Technology, Danvers, MA, USA), anti-p44/42 MAPK mouse antibody (1:500, #4696; Cell Signalling Technology) anti-AKT mouse antibody (1:250, #2920; Cell Signalling Technology).

Immunostaining was carried out using a Discovery XT Automated IHC Stainer with a Ventana DABMap Detection Kit (No. 760–124; Ventana Medical System, Oro Valley, AZ, USA). Each step of the Ventana DABMap Detection Kit procedure was optimized for the Discovery XT instrument; the conditions, such as the dilutions of each antibody, were established before the measurements. In all cases, the antigen was activated by heating.

Fisher’s exact test was used for statistical analysis, and the level of significance was *P* < 0.05.

### Verification of a novel 3D culture system using Cellbed

#### Characteristics of non-woven silica fibre sheet (Cellbed)

**Fig. 8:**
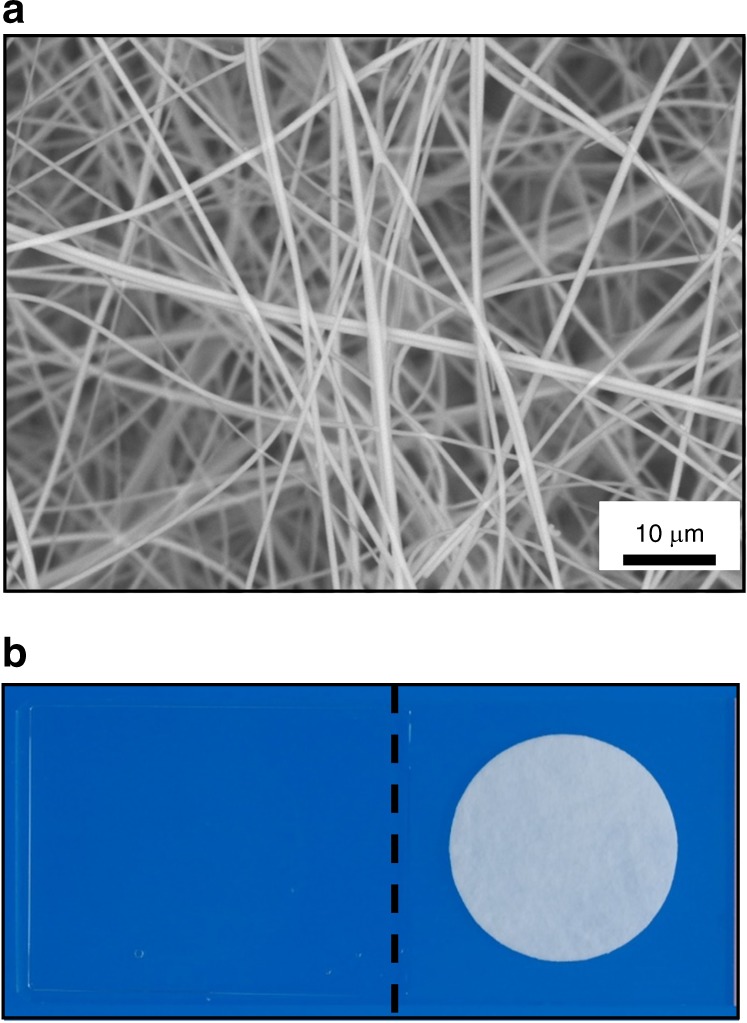
Characteristics of Cellbed. **a** Silica fibre aggregate fixed in the form of a sheet; the average thickness of the silica fibres was approximately 1 μm (scale bar: 10 μm). **b** Cellbed was rendered transparent by enclosure in a mounting medium with a refractive index of 1.46 (left: Cellbed after mounting, right: Cellbed before mounting)

In this study, a non-woven silica fibre sheet (Cellbed) was used as the 3D culture support. Cellbed was supplied by Japan Vilene Co., Tokyo, Japan. Cellbed is a fibre aggregate produced by consolidating continuously produced silica fibres in a sheet form, forming an ultrafine non-woven silica glass fibre sheet (200 μm thick). The average thickness of the silica fibres was approximately 1 μm, and the mean flow pore diameter measured by the bubble point test was 7 μm–8 μm. Cellbed is opaque in air; therefore, it is difficult to observe the cells during cell culture. However, after staining, the cells can be observed using a mounting medium with the same refractive index (1.46) as the silica fibres (Fig. 8a, b).

#### Cell culture

In the 3D cell culture system, we spread Cellbed on dishes or well plates and cultured the cells on it. The other conditions were mostly the same between the 2D and 3D culture system. However, we could prolong the duration of cell culture in the 3D culture system for up to 4 weeks. Two tongue cancer cell lines, HSC-3 and HSC-4, were purchased from the JCRB Cell Bank (Tokyo, Japan). The culture medium used was Dulbecco’s modified Eagle’s medium (Nacalai Tesque, Kyoto, Japan) containing 10% foetal bovine serum (Sigma-Aldrich, St. Louis, MO, USA) and 1% antibiotic-antimycotic solution (Invitrogen, Carlsbad, CA, USA). Cell culture was carried out at 37 °C in the presence of 5% CO_2_.

#### HE staining

Seventy-two hours after seeding the cells (HSC-3 and HSC-4: 1× 10^5^ cells per mL) into a 12-well plate on glass coverslips and Cellbed, the cells were washed with phosphate buffered saline (PBS) and then fixed for 30 min in PBS containing 10% formalin. Cell lines cultured on Cellbed were embedded in paraffin. Serial 5-μm sections were histologically evaluated by HE staining. Cells cultured on glass coverslips were stained with HE without being embedded in paraffin.

#### Cell growth assay

The MTT assay was used to evaluate the proliferation and growth of 2D- and 3D-cultured cells. The experiment was repeated three times. Cells were seeded in 96-well plates (2D-cultured HSC-3 and HSC-4: 1 × 10^4^ cells per mL), and MTT solution (0.25 mg•mL^-1^) was added to the medium after incubation for 48, 72 or 96 h. Formazan crystals were dissolved in dimethyl sulfoxide, and the absorption was measured at 570 nm using an Infinite 200 microplate reader (TECAN, Kawasaki, Japan). Then, 3D-cultured cells were seeded in Cellbed® 96-well plates (3D-cultured HSC-3 and HSC-4: 5 × 10^4^ cells per mL) and were analysed in the same way as 2D-cultured cells.

#### Immunohistochemical staining of HSC-4 cells cultured on Cellbed

Cells cultured on Cellbed were embedded in paraffin. Serial 5-μm sections were histologically evaluated by Ki-67 staining. HSC-4 cells cultured on Cellbed were stained in the same manner as the human tongue cancer tissue. Immunohistochemical staining was performed using an anti-Ki67 rabbit monoclonal antibody (cat. # ab16667; Abcam plc, Cambridge, UK).

#### Toluidine blue staining and electron microscopy of HSC-4 cells 3D-cultured for approximately 4 weeks

The sample used was HSC-4 cells cultured on Cellbed for approximately 4 weeks. Cell culture was performed in a 96-well plate; during the 4-week culture period, the size of the container was gradually increased to supply the cells with an adequate quantity of culture medium. At the end of the cultures, culture was carried out in a 16-cm dish.

Samples were prepared for imaging by Tokai Electron Microscopy Inc. (Nagoya, Japan). The samples were fixed with 2% paraformaldehyde and 2% glutaraldehyde in 0.1 mol•L^-1^ phosphate buffer (PB; pH 7.4). Thereafter, they were fixed with 2% glutaraldehyde in 0.1 mol•L^-1^ PB at 4 °C overnight. The samples were post-fixed with 2% osmium tetroxide in 0.1 mol•L^-1^ PB at 4 °C for 90 min. Furthermore, the samples were infiltrated with propylene oxide (PO) two times for 20 min each and then placed into a 70:30 mixture of PO and resin (Quetol-812; Nissin EM Co., Tokyo, Japan) for 1 h. The cap of the tube was kept open, and PO was volatilized overnight. The polymerized resins were cut into semithin sections of 1.5 μm with glass knives using an ultramicrotome (Ultracut UCT; Leica, Vienna, Austria) and stained with 0.5% toluidine blue. The polymerized resins were cut into ultrathin sections of 70 nm with a diamond knife using an ultramicrotome (Ultracut UCT; Leica, Vienna, Austria), and the sections were mounted on copper grids. The resins were stained with 2% uranyl acetate at room temperature for 15 min and then were secondary-stained with a lead stain solution (Sigma-Aldrich, Tokyo, Japan). The grids were observed under a transmission electron microscope (JEM-1400Plus; JERO Ltd., Tokyo, Japan) at an acceleration voltage of 80 kV.

### The roles of ERK and ezrin in cancer development

#### Ezrin siRNA

Ezrin siRNA (5′-CCCUUGGACUGAAUAUUUAUGAGAA-3′) and a non-silencing control siRNA were purchased from Invitrogen. Following the manufacturer’s protocol, 48 h after seeding the cells (HSC-3 and HSC-4: 1 × 10^5^ cells per mL) into a 12-well plate, the cells were transfected using Lipofectamine RNAiMAX reagent (Invitrogen). Transfected cells were designated as siEzrin cells.

#### ERK inhibitor

The ERK inhibitor used was FR180204 (Merck CalBiochem, Darmstadt, Germany). Forty-eight hours after seeding the cells (HSC-3 and HSC-4: 10 × 10^4^ cells per mL) into a 12-well plate, the culture medium was replaced with medium containing FR180204 (30 or 50 μmol•L^-1^). Six hours after replacing the culture medium, the protein was recovered for western blotting. Cells cultured in the presence of ERK inhibitor were designated as FR cells.

#### Western blotting

The experiment was repeated three times. The Cellbed was flushed (9 100 g, 4 °C) to remove moisture after washing with PBS, was then immersed in lysis buffer [50 mmol•L^-1^ Tris–HCl (pH 7.4), 150 mmol•L^-1^ sodium chloride, 0.5 mmol•L^-1^ EDTA, 0.09 U•mL^-1^ aprotinin, 1 mg•mL^-1^ pepstatin, 10 mmol•L^-1^ phenylmethylsulfonyl fluoride and 1 mg•mL^-1^ leupeptin], and incubated for 30 min on ice. The Cellbed and lysis buffer were then transferred to a spin column and centrifuged (13 000 g, 4 °C, 10 min). The protein concentration of the lysate collected in the centrifuge tube was measured by the BCA assay. The protein lysate (5 μg per lane) was separated on a 4%–12% sodium dodecyl sulfate-polyacrylamide gel (NuPAGE, Invitrogen) and then transferred onto a poly(vinylidene difluoride) membrane (Invitrogen). Before incubation with primary antibodies, the film was blocked with TBS-T [20 mmol•L^-1 ^Tris–HCl (pH 7.5), 8 g•L^-1^ sodium chloride, and 0.1% Tween 20] containing 4% dried skim milk.

As primary antibodies, anti-ezrin mouse antibody (1:1 000, ab4069; Abcam, Cambridge, UK), anti-β-actin mouse monoclonal antibody (1:1 000, sc-47778; Santa Cruz Biotechnology), anti-phospho-p44/42 MAPK (ERK1/2) rabbit antibody (1:1 000, #4370; Cell Signaling Technology), anti-cortactin rabbit antibody (1:20 000, ab81208; Abcam), and anti-phospho-cortactin (Tyr421) rabbit antibody (1:500, #4569; Cell Signaling Technology) were employed.

As secondary antibodies, goat peroxidase-conjugated anti-rabbit IgG (#L3012; Signalway Antibody, TX, USA) and goat peroxidase-conjugated anti-mouse IgG (#AP124P; Millipore, Billerica, MA, USA) were employed. β-Actin was used as the positive control. Furthermore, t-ERK was also used as the positive control in the experiment using FR180204. Proteins were visualized using a horseradish peroxidase substrate and scanned with an enhanced chemiluminescence system (Las 4000; Fuji Film, Tokyo, Japan). The band intensity was normalized against β-actin.

#### Immunofluorescence

Forty-eight hours after seeding the cells (HSC-3 and HSC-4: 1 × 10^5^ cells per mL) into a 12-well plate, the culture medium was replaced with a medium containing FR180204 (50 μmol•L^-1^). Twenty-four hours after replacing the culture medium, the cells were fixed for 30 min in PBS containing 10% formalin. After membrane permeation treatment for 1 h with PBS containing 0.1% Tween 20, blocking with Histofine Blocking Reagent II (Nichirei Biosciences Inc., Tokyo, Japan) and 10% normal goat serum was carried out for 30 min. The cells were incubated at 4 °C overnight before reacting with the primary antibodies. The primary antibodies used were anti-cortactin rabbit antibody (1:1 000, ab81208; Abcam) and anti-ezrin mouse antibody (1:100, ab4069; Abcam). Cells were then incubated with the secondary antibodies for 1 h at room temperature. The secondary antibody used was Alexa Fluor 488 or 594-labelled anti-mouse IgG antibody or rabbit IgG antibody (Invitrogen). Rhodamine–phalloidin (Invitrogen) was employed for labelling actin. DAPI (4′,6-Diamidino-2-phenylindole dihydrochloride) (Wako Pure Chemical Industries, Osaka, Japan) was employed for nuclear labelling, and ProLong® Diamond Antifade Mountant (Life Technologies, Carlsbad, CA, USA) was used for mounting. A confocal laser scanning microscope (FV-1000D IX-81; Olympus, Tokyo, Japan) was used to visualize the cells.
